# The autotransporter protein BatA is a protective antigen against lethal aerosol infection with *Burkholderia mallei* and *Burkholderia pseudomallei*

**DOI:** 10.1016/j.jvacx.2018.100002

**Published:** 2018-12-22

**Authors:** Eric R. Lafontaine, Zhenhai Chen, Maria Cristina Huertas-Diaz, Jeremy S. Dyke, Tomislav P. Jelesijevic, Frank Michel, Robert J. Hogan, Biao He

**Affiliations:** a Department of Infectious Diseases, University of Georgia College of Veterinary Medicine, Athens, GA 30602, USA; b Department of Veterinary Biosciences and Diagnostic Imaging, University of Georgia College of Veterinary Medicine, Athens, GA 30602, USA

**Keywords:** Glanders, Melioidosis, Lethal aerosol challenge, PIV5 vaccine vector, Tier 1 select agent, Autotransporter protective antigen

## Abstract

**Background:**

*Burkholderia mallei* and *Burkholderia pseudomallei* are the causative agents of glanders and melioidosis, respectively. There is no vaccine to protect against these highly-pathogenic and intrinsically antibiotic-resistant bacteria, and there is concern regarding their use as biological warfare agents. For these reasons, *B. mallei* and *B. pseudomallei* are classified as Tier 1 organisms by the U.S. Federal Select Agent Program and the availability of effective countermeasures represents a critical unmet need.

**Methods:**

Vaccines (subunit and vectored) containing the surface-exposed passenger domain of the conserved *Burkholderia* autotransporter protein BatA were administered to BALB/c mice and the vaccinated animals were challenged with lethal doses of wild-type *B. mallei* and *B. pseudomallei* strains via the aerosol route. Mice were monitored for signs of illness for a period of up to 40 days post-challenge and tissues from surviving animals were analyzed for bacterial burden at study end-points.

**Results:**

A single dose of recombinant Parainfluenza Virus 5 (PIV5) expressing BatA provided 74% and 60% survival in mice infected with *B. mallei* and *B. pseudomallei*, respectively. Vaccination with PIV5-BatA also resulted in complete bacterial clearance from the lungs and spleen of 78% and 44% of animals surviving lethal challenge with *B. pseudomallei*, respectively. In contrast, all control animals vaccinated with a PIV5 construct expressing an irrelevant antigen and infected with *B. pseudomallei* were colonized in those tissues.

**Conclusion:**

Our study indicates that the autotransporter BatA is a valuable target for developing countermeasures against *B. mallei* and *B. pseudomallei* and demonstrates the utility of the PIV5 viral vaccine delivery platform to elicit cross-protective immunity against the organisms.

## 1 Introduction

*Burkholderia pseudomallei* and *Burkholderia mallei* are closely-related bacteria that cause fatal infections in humans and animals. *Burkholderia pseudomallei* is commonly found in wet soils of countries bordering the equator and causes the emerging global disease melioidosis [1], [2], [3]. *Burkholderia mallei* is a host-adapted clone of *B. pseudomallei* that does not persist in the environment outside of its natural equine reservoir. The organism causes the extremely contagious and incapacitating zoonotic disease glanders, which is a re-emerging biosecurity threat closely monitored by the World Organization for Animal Health [4], [5], [6].

Comparative analyses indicate that *B. mallei* evolved from *B. pseudomallei* through genomic reduction and the genes retained by *B. mallei* have an average identity of 99% with their *B. pseudomallei* orthologs [7], [8], [9], [10]. The clinical and pathological manifestations of disease caused by the organisms are also strikingly similar. In humans, infection typically occurs through punctured skin or the respiratory route, and the most common manifestations are life-threatening pneumonia and bacteremia [1], [6], [11], [12]. Pathogenicity involves the coordinated expression of many virulence factors that support extracellular and intracellular replication of bacteria as well as the seeding of deep tissues where the organisms form lesions that are difficult to eliminate [13], [14], [15], [16].

Glanders and melioidosis are difficult to diagnose and require prolonged therapy with low success rates due in large part to intrinsic resistance of the organisms to antibiotics [17], [18]. No vaccine exists to protect humans or animals and there is concern regarding adversarial use given that *B. mallei* has previously been utilized as a biological warfare agent [6]. For these reasons, the U.S. Federal Select Agent Program classifies *B. mallei* and *B. pseudomallei* as Tier 1 organisms and the availability of medical countermeasures is considered a critical unmet need. The genetic, biochemical, and virulence similarities between *B. mallei* and *B. pseudomallei*, and the marked resemblance of the disease they cause, supports the feasibility of developing countermeasures that protect against both organisms. This belief is supported by studies demonstrating that passive transfer of antibodies against *in vivo* expressed *Burkholderia* antigens provides protection in mice against lethal challenge with *B. pseudomallei* and *B. mallei* [19], and subunit vaccines containing *Burkholderia* exopolysaccharides [20], [21] and outer membrane vesicles [22], [23] elicit cross-species immunity in glanders and melioidosis infection models.

A number of experimental vaccines have been developed but none achieve complete protection and sterile immunity [24], [25], [26]. Best-in-class vaccines afford protection against lethal challenge but do not prevent persistence of the organisms; animals develop lesions with high tissue burden and succumb to chronic infection despite possessing robust humoral and cellular immunity against *B. pseudomallei* and *B. mallei*. This failure to eliminate infection is a major obstacle in the field and emphasizes the need to expand the current pool of high value *Burkholderia* antigens for vaccine generation and develop efficacious delivery platforms [24], [25], [26].

With this in mind, our group previously identified the protein BatA as a potential target for devising countermeasures [19]. We showed that BatA is an autotransporter protein located on the bacterial surface and thus readily accessible for recognition by the immune system. We demonstrated that the *batA* gene is present in the genome of all sequenced *B. mallei* and *B. pseudomallei* strains and that the encoded protein is highly conserved at the amino acid level (99–100%). We demonstrated that BatA aids in intracellular survival, is expressed *in vivo*, and elicits production of antibodies during infection. Furthermore, we discovered that a *B. mallei* mutant lacking expression of BatA is attenuated in virulence using a mouse model. Autotransporter proteins represent one of the largest class of virulence factors in Gram-negative organisms and contribute a wide range of phenotypes, such as host cell adhesion, biofilms, and serum resistance [27], [28], [29], [30]. Many studies have demonstrated that autotransporters are immunoprotective antigens [31], [32], [33], [34], [35], [36], and the inclusion of the host cell adhesion autotransporter proteins NadA and Pertactin in licensed subunit vaccines for *Neisseria meningitidis* (Bexsero) and *Bordetella pertussis* (Daptacel, Infanrix, Boostrix, and Adacel), respectively, underscores their value for devising medical intervention strategies. Hence, BatA displays many attributes of a strong candidate for developing countermeasures and targeting the protein may interfere with the ability of *B. mallei* and *B. pseudomallei* to establish themselves in a host, persist, and cause infection.

Parainfluenza virus 5 (PIV5) is a well-studied paramyxovirus and excellent vector for vaccine development [37]. It is safe, inexpensive to produce, and has been previously shown to be efficacious as the backbone of vaccines for multiple high impact agents including influenza [38], rabies [39], and RSV [40]. The platform has also been shown to elicit protection against *Mycobacterium tuberculosis* [41], which shares many pathogenicity traits with *B. mallei* (high aerosol infectivity, replicates extracellularly and within host cells, disseminates to deep tissues and forms chronic lesions). The goal of the present study was to test the vaccinogenic potential of BatA and develop a vaccine delivery platform for the antigen using PIV5.

## 2 Materials and methods

### 2.1 Cells and culture conditions

BHK21 and BSR-T7 cells were cultured in DMEM supplemented with 10% Tryptose Phosphate Broth (ThermoFisher Scientific) and 10% fetal bovine serum (FBS). The medium for BSR-T7 cells also contained 400 µg/mL of G418 (ThermoFisher Scientific). MDBK cells were grown in DMEM containing 10% FBS. All cultures were maintained at 37 °C with 5% CO_2_.

### 2.2 Purification of BatA protein

*Escherichia coli* TUNER (EMD Millipore) carrying plasmids pHisBatA and pGSTBatA was used to produce recombinant forms of the surface-exposed domain of the BatA protein (residues 30–307) joined to His and GST tags, respectively. Both proteins were purified as reported [19]. A Pierce LAL Chromogenic Endotoxin Quantitation Kit (ThermoFisher Scientific) was used to measure the amount of endotoxin in protein preparations.

### 2.3 Generation of PIV5 expressing BatA protein

Portion of the *B. mallei* ATCC 23,344 *batA* gene (locus tag BMA1647) was synthesized with optimized codon usage for human cells (GenScript custom gene synthesis services). The gene fragment, which specifies residues 30–307 of the BatA protein, was inserted between the SH and HN genes of PIV5 in a plasmid containing the PIV5 genome. The sequence of the resulting virus, PIV5-BatA, was verified by RT-PCR sequencing. PIV5 expressing *Mycobacterium tuberculosis* protein Ag85B (PIV5-Tb) was used as control in efficacy studies and has been described elsewhere [41].

### 2.4 Growth curve and plaque assay

MDBK cells were infected with PIV5 viruses at a MOI of 0.1 and supernatants were collected daily for a period of 5 days post-infection. Virus titers were determined by plaque assay using BHK21 cells as previously outlined [41].

### 2.5 Western blotting

Antigen preparations were resolved by SDS-PAGE, transferred to PVDF membranes (EMD Millipore), and probed with antibodies as published [28]. In some experiments, antigens resolved by SDS-PAGE were stained with SimplyBlue™ SafeStain (ThermoFisher Scientific). The mouse antibody His-Tag (EMD Millipore) was used in experiments with His-tag BatA protein. Murine antibodies against a peptide encompassing BatA residues 208–221 (GenScript custom polyclonal antibody production services) were used in experiments with PIV5-BatA virus. Goat anti-mouse antibodies conjugated to HRP (SouthernBiotech) were utilized for detection.

### 2.6 Preparation of *B*. *Mallei* and *B*. *Pseudomallei* cultures

The wild-type strains *B. mallei* ATCC 23,344 [10] and *B. pseudomallei* K96243 [9] were used as challenge agents in efficacy studies. The *B. mallei batA* KO live attenuated strain (LAS) [19] was used as protection benchmark. *Burkholderia mallei* was cultured on agar plates for 40 h at 37 °C using brucella medium (BD) supplemented with 5% glycerol. *Burkholderia pseudomallei* was cultured on agar plates for 20 h at 37 °C using tryptic soy medium (BD).

### 2.7 Animal vaccination and challenge studies

Female BALB/c mice (6–8 weeks) were purchased from Envigo. Vaccination with PIV5 viruses was performed intranasally. Mice were anesthetized intraperitoneally with 250 mg/kg of tribromoethanol (SIGMA-ALDRICH). After confirming loss of pedal reflex, animals were held in supine position and 5–10 µL droplets of virus suspension were delivered to the nostrils (total volume administered of 50 µL, dose = 10^7^ PFU). Six weeks later, mice were challenged using a Microsprayer device as previously reported [42]. Infected animals were monitored daily, food and water were provided *ad libitum*, and humane endpoints were strictly observed. At study end-points, survivors were euthanized and tissues were harvested, homogenized, serially-diluted, and plated onto agar medium to determine bacterial burden. Studies were focused on determining bacterial burden in the lungs and spleen, which are well-characterized target organs for *B. mallei* and *B. pseudomallei*. Age- and weight-matched naïve animals were used as unvaccinated controls and mice vaccinated with *batA* KO LAS served as protection benchmark. The latter were administered 10^4^ CFU of LAS and back-challenged with wild-type agents 30–45 days post-vaccination as described by Zimmerman et al. [19]. Challenge doses of 8000 (10 LD_50_) and 300 (5 LD_50_) CFU of *B. mallei* ATCC 23,344 and *B. pseudomallei* K96243 were used, respectively.

Vaccination with His-tag BatA protein was performed intranasally as described above for PIV5 viruses with some modifications. Specifically, mice were administered 3 vaccine doses, 21 days apart. At the same time, the animals were also administered the vaccine subcutaneously. Each dose (intranasal and subcutaneous) consisted of 10 µg His-tag BatA mixed in a ratio of 1:1 with RECOMBITEK Lyme (rLyme, MERIAL). The latter is a liquid suspension of the *Borrelia bugdorferi* outer surface protein A, a TLR-2 agonist with proven efficacy as adjuvant [43]. This vaccination approach (intranasal and subcutaneous) was used to stimulate mucosal and systemic immune responses. Thirty days after the last boost, animals were challenged with *B. mallei* ATCC 23,344 using a Microsprayer. Age- and weight-matched control mice were administered rLyme alone on the same schedule. Mice vaccinated with *batA* KO LAS and back-challenged with wild-type organisms were used as protection benchmark.

### 2.8 Elisa

Duplicate wells of Immulon 2HB plates (ThermoFisher Scientific) were coated with antigens (GST-tag BatA, PIV5) and the antibody reactivity of serum samples to these antigens was determined according to the method of Zimmerman et al. [28].

### 2.9 Elispot

The spleens of vaccinated mice were collected, processed into single cell suspensions, splenocytes were stimulated with His-tag BatA protein, and the number of cytokine-secreting cells were determined as reported by Chen and colleagues [41].

### 2.10 Data analysis

Survival data were analyzed with the Kaplan-Meier method, and the Log-rank Mantel-Cox and Gehan-Breslow-Wilcoxon tests were used to perform statistics. Other comparisons were made using Student’s *t* and Mann-Whitney tests (ELISA data) as well as Chi-square (and Fisher exact) test (presence or absence of bacteria in the lungs and spleen of mice surviving challenge). All analyses were performed using the GraphPad Prism software.

### 2.11 Compliance and ethics statements.

The University of Georgia’s Institutional Biosafety Committee approved the experiments in this study. All experiments with live *B. mallei* and *B. pseudomallei* were performed inside a class II biosafety cabinet inside a biosafety level 3 (BSL3) laboratory in compliance with the rules and regulations of the U. S. Federal Select Agent Program. Infected animals were housed in an Innorack IVC dual-HEPA-filtered ventilated system (Innovive) located in an animal BSL3 (ABSL3) laboratory.

The University of Georgia’s Institutional Animal Care and Use Committee approved the animal experiments in this study. All animal experiments were performed in strict accordance with the recommendations in the Guide for the Care and Use of Laboratory Animals of the National Institutes of Health. Every effort was made to minimize animal suffering.

## 3 Results

### 3.1 Vaccination with purified BatA protein provides protection against *B*. *Mallei* challenge

**Fig. 1:**
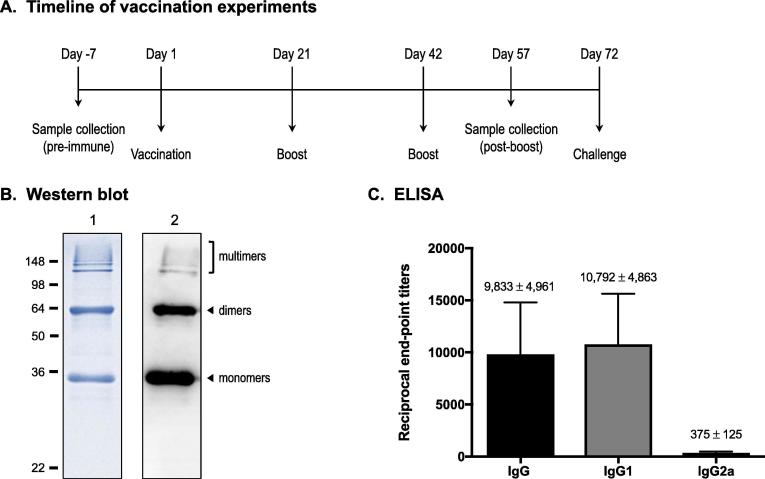
*Western blot and ELISA analysis of serum from mice immunized with BatA protein*. (A) Timeline of vaccination experiments. (B) Approximately 5 µg of His-tag BatA was resolved by SDS-PAGE. The resolved protein was stained with Coomassie (lane 1) or transferred to a PVDF membrane for western blot analysis with an antibody specific for the His tag (lane 2). Antibodies conjugated to horseradish peroxidase (HRP) were used for detection, and protein bands were visualized by chemiluminescence. Molecular weight markers are shown to the left in kilodaltons. (C) Individual serum samples from mice immunized with His-tag BatA protein were serially diluted, placed in duplicate wells of plates coated with GST-tag BatA protein, and tested by standard ELISA. Alkaline-phosphatase-conjugated goat anti-mouse antibodies specific for IgG1, IgG2a, or for the heavy and light chains of IgG, were used for detection. The data are expressed as the mean (±standard error) end point titer of samples from *n* = 6 animals. Individual titers were determined using pre-immune samples as background, and correspond to the highest immune serum dilutions giving ELISA values greater than the mean value of pre-immune serum plus 3 standard deviations. The endotoxin level of the His-tag BatA protein preparation used to immunize mice was calculated to be 0.8 EU/mL.

Our group previously reported that the N-terminus of BatA is exposed to the bacterial surface [19]. With this in mind, we vaccinated mice with a recombinant form of the BatA surface-located domain (predicted molecular weight of 30-kDa). Fig. 1B shows SDS-PAGE and western blot analyses of the protein preparation used to vaccinate. The ELISA data in Fig. 1C demonstrate that mice produced BatA-specific antibodies with a Th2 bias IgG1/IgG2a ratio of 28.8.

**Fig. 2:**
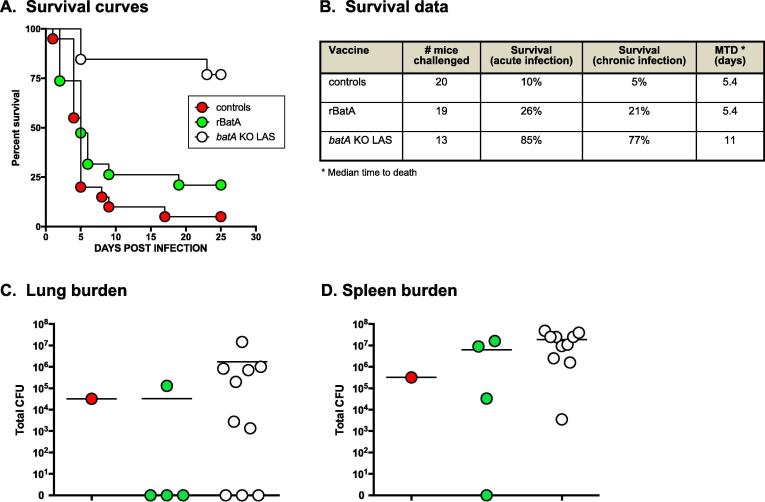
*Vaccination with BatA protein provides protection against challenge with a lethal dose of* B. mallei *ATCC23344*. Mice vaccinated with BatA protein mixed with adjuvant (rBatA), adjuvant only (controls), and *batA* KO LAS (benchmark of protection) were challenged with 10LD_50_ of wild-type *B. mallei* ATCC 23,344 and monitored daily for clinical signs of illness and morbidity. (A) Kaplan-Meier survival curves. (B) Survival data during the acute (days 1–10 post-challenge) and chronic (days 11–25 post-challenge) phases of infection. (C and D) At study end-points, tissues collected from survivors were homogenized, diluted, and spread onto agar plates to determine bacterial loads. The symbols show data for individual animals; horizontal lines represent the mean total CFU for each group. The experiments were performed on 3 separate occasions; the graphs and table show cumulative results. The survival curves were compared using the Log-rank Mantel-Cox and Gehan-Breslow-Wilcoxon tests and found to be significantly different with values of *P* < 0.0001 and *P* = 0.0003, respectively. The curves for the adjuvant controls and *batA* KO LAS groups were found to be significantly different with *P* values < 0.0001 in both tests. The curves for the rBatA and *batA* KO LAS groups were found to be significantly different with *P* values of 0.0015 (Log-rank Mantel-Cox) and 0.0022 (Gehan-Breslow-Wilcoxon).

After confirming development of an immune response against BatA, mice were challenged with the *B. mallei* wild-type strain ATCC 23344. As shown in Fig. 2A and B, the BatA subunit vaccine provided 26% protection against mortality during acute infection and 21% of challenged animals survived the duration of the experiments. Control mice administered adjuvant alone showed 10% and 5% survival during acute infection and at study endpoints, respectively. Age- and weight-matched mice vaccinated with the *B. mallei batA* KO live attenuated strain (LAS) were used as protection benchmark and consistent with our previously published findings [19], the LAS provided high levels of protection against death during acute and chronic infection.

At study endpoints, tissues were collected from survivors and the bacterial burden was determined. No bacteria were detected in the lungs of *n* = 3 out of 4 survivors given the BatA subunit vaccine (Fig. 2C). One mouse from the BatA subunit vaccine group also showed complete clearance from the spleen (Fig. 2D). One animal administered adjuvant alone survived the duration of the experiment and was colonized with *B. mallei* in the lungs and spleen (Fig. 2C and 2D, respectively). No bacteria were detected in the lungs of *n* = 3 out of 10 survivors vaccinated with *batA* KO LAS (Fig. 2C). All surviving mice from the LAS vaccine group were colonized with wild-type *B. mallei* in the spleen (Fig. 2D). Taken together, these data indicate that vaccination with BatA protein elicits limited protection against lethal challenge with *B. mallei* and resulted in 75% of surviving animals with no detectable organisms in the lungs.

### 3.2 Generation of a vector-based vaccine delivery system for BatA

**Fig. 3:**
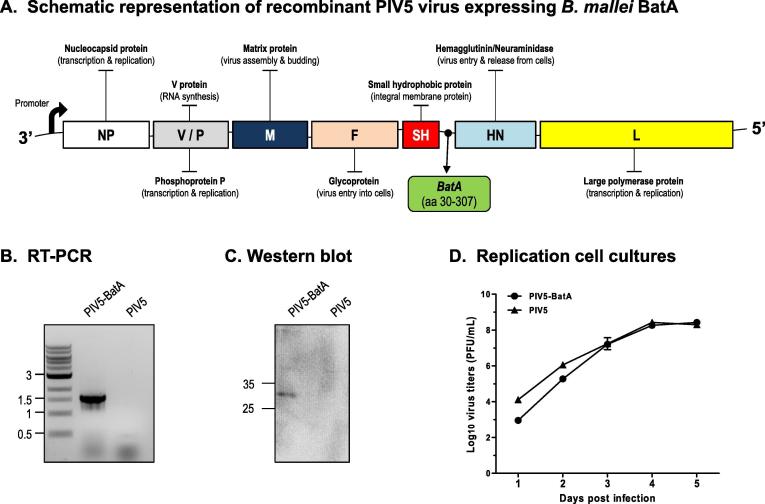
*Generation of recombinant PIV5 virus expressing* B. mallei *BatA*. (A) Schematic representation of the 8 gene products encoded by the PIV5 genome. The gene fragment specifying residues 30–307 of the *B. mallei* ATCC 23,344 BatA protein was cloned between the SH and HN genes. The resulting construct was transfected into cells for plaque purification and amplification of recombinant PIV5-BatA virus. (B) Viral RNA extracted from the supernatants of cell cultures infected with recombinant PIV5-BatA and wild-type PIV5 viruses was analyzed by RT-PCR using the primers batAF and 7169R, which are specific for the *batA gene* and PIV5 HN, respectively. (C) Equivalent protein amount of lysates from cells infected with PIV5-BatA and PIV5 viruses were analyzed by western blotting with BatA-specific antibodies. Molecular mass markers are shown on the left in kilodaltons. (D) MDBK cells were infected with PIV5-BatA and PIV5 viruses at an MOI of 0.1. Aliquots of supernatant from the infected cell cultures were collected at 24 h intervals and titers were determined by plaque assay using BHK21 cells. The results are expressed as the mean (±standard deviation) Log10 PFU/mL. The experiment was performed on 2 separate occasions and the graph shows cumulative results.

Based on the data demonstrating that vaccination with purified BatA protein modestly protects against *B. mallei* lethal aerosol challenge, we hypothesized that delivering the antigen with a viral vector vaccine platform would evoke superior protection. To test this, a gene fragment corresponding to the surface-exposed domain of BatA was cloned between the SH and HN genes of PIV5 as illustrated in Fig. 3A. To verify cloning of the gene fragment in its intended location, RNA was extracted from the supernatant of cell cultures infected with recombinant PIV5-BatA virus and analyzed by RT-PCR using a forward oligonucleotide primer specific for *batA* in combination with a reverse primer that binds near the middle of the PIV5 HN gene. As control, viral RNA was obtained from parallel cultures infected with wild-type PIV5 virus and analyzed in the same manner. The data in Fig. 3B show that a PCR product of 1.5-kb was amplified from cells infected with PIV5-BatA whereas no amplicon was detected in the PIV5 extracts, as expected. The genome of PIV5-BatA was also sequenced to confirm that no unwanted mutations were introduced during the cloning process (data not shown).

To determine if the virus specifies expression of the BatA protein, lysates from cells infected with PIV5-BatA and wild-type PIV5 viruses were analyzed by Western blotting. As shown in Fig. 3C, BatA-specific antibodies did not react with the PIV5 lysate but bound to a protein of 30-kDa in cells infected with PIV5-BatA. The replication rates of the viruses were also compared over a period of 5 days (Fig. 3D). PIV5-BatA displayed slightly delayed growth kinetics during the first 48 h post-infection compared to wild-type PIV5 but grew to comparable titers thereafter. Thus, BatA expression does not impair the fitness and infectivity of PIV5 *in vitro*.

### 3.3 Vaccination with PIV5-BatA provides protection against challenge with *B*. *Mallei* and *B*. *Pseudomallei*

**Fig. 4:**
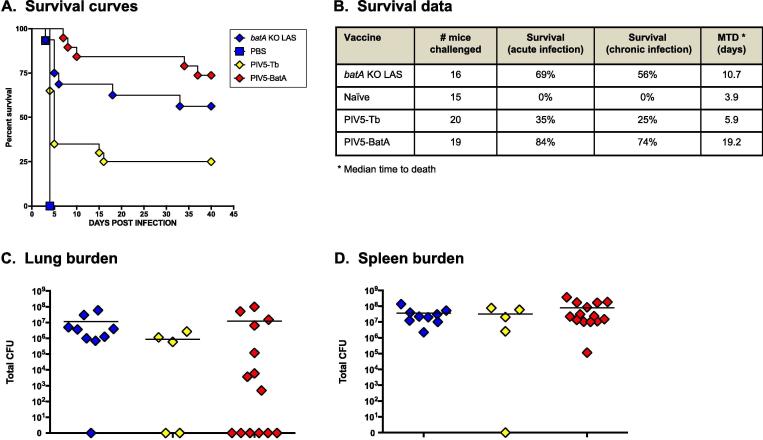
*Vaccination with PIV5-BatA provides protection against challenge with a lethal dose of* B. mallei *ATCC23344.* Mice vaccinated with PIV5 viruses were challenged with 10 LD_50_ of wild-type *B. mallei* ATCC23344 and monitored daily for clinical signs of illness and morbidity. Controls consisted of age- and weight-matched naïve mice (negative control group) and mice vaccinated with *batA* KO LAS (benchmark of protection group). (A) Kaplan-Meier survival curves. (B) Survival data during the acute (days 1 through 10 post-challenge) and chronic (days 11 through 40 post-challenge) phases of infection. (C and D) At study end-points, tissues collected from survivors were homogenized, diluted, and spread on agar plates to determine bacterial loads. The symbols show data for individual animals; horizontal lines represent the mean total CFU for each group. The experiments were performed on 2 separate occasions; the graphs and table show cumulative results. The survival curves were compared using the Log-rank Mantel-Cox and Gehan-Breslow-Wilcoxon tests and found to be significantly different with *P* values < 0.0001 in both tests. The curves for the PIV5-BatA and PIV5-Tb groups were found to be significantly different with *P* values of 0.0005 (Log-rank Mantel-Cox) and 0.0001 (Gehan-Breslow-Wilcoxon). The survival curve for the PBS group was found to be significantly different from that of the PIV5-Tb group, the PIV5-BatA group, and the *batA* KO LAS group (*P* < 0.0001 in both tests and for all pairwise comparisons). The curves for the PIV5-Tb and *batA* KO LAS groups were significantly different with *P* values of 0.0310 (Log-rank Mantel-Cox) and 0.0190 (Gehan-Breslow-Wilcoxon).

To determine if delivering BatA with a vector-based platform elicits protection, mice were immunized with PIV5-BatA and, 45 days later, challenged with *B. mallei* ATCC 23344. Control groups consisted of age- and weight-matched naïve mice, mice vaccinated with *batA* KO LAS, and animals immunized with recombinant PIV5-Tb virus specifying expression of the *M. tuberculosis* protein Ag85B. As shown in Fig. 4A and 4B, naïve controls all succumbed to infection by day 10 post-challenge, while LAS vaccination provided 69% and 56% survival during acute and chronic infection, respectively. Immunization with PIV5-BatA afforded 84% and 74% protection against death during acute and chronic infection, respectively, and increased the median time to death to 19 days (from 4 to 6 days in negative control groups). We found that vaccination with PIV5-Tb control virus provided some protection against death (35% survival during acute infection, 25% during chronic), presumably by priming pro-inflammatory innate responses in the lungs (mice are immunized intranasally with live virus, which results in recruitment of inflammatory cells). Similar non-specific protection (*i.e.* “adjuvant effect”) has previously been reported in animals treated with viruses and adjuvants prior to experimental challenge with infectious agents including *B. mallei* and *B. pseudomallei* [44], [45], [46].

At study end-points, we determined tissue burden in survivors and discovered that no bacteria could be detected in the lungs of *n* = 6 out of 14 mice vaccinated with PIV5-BatA (Fig. 4C) and that these animals were all colonized with *B. mallei* in the spleen (Fig. 4D). Two out of the 5 surviving mice given the PIV5-Tb control virus had no detectable bacteria in the lungs (Fig. 4C) and one animal from the group completely cleared *B. mallei* from the spleen (Fig. 4D). All survivors from the benchmark LAS vaccine group were colonized with wild-type *B. mallei* in the spleen (Fig. 4D) and one mouse from the cohort had no detectable bacteria in the lungs (Fig. 4C). Taken together, the data demonstrate that a single dose of PIV5-BatA provides excellent survival against *B. mallei* lethal aerosol challenge.

**Fig. 5:**
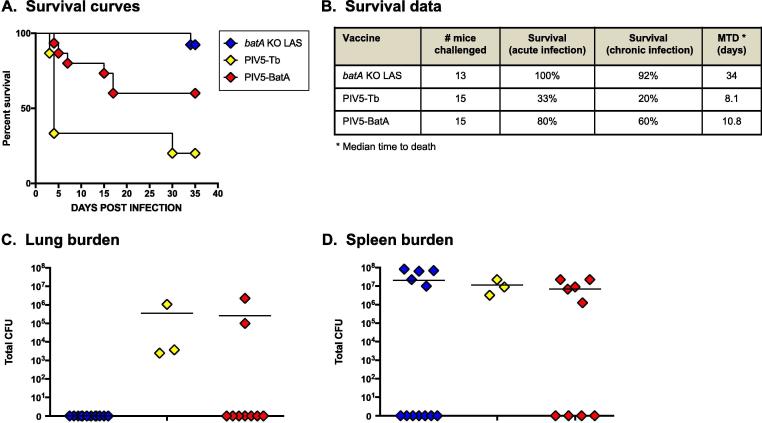
*Vaccination with PIV5-BatA provides protection against challenge with a lethal dose of B*. *pseudomallei K96243.* Mice vaccinated with PIV5 viruses were challenged with 5 LD_50_ of wild-type *B. pseudomallei* strain K96243 and monitored daily for clinical signs of illness and morbidity. Controls consisted of age- and weight-matched mice vaccinated with *batA* KO LAS (benchmark of protection group). (A) Kaplan-Meier survival curves. (B) Survival data during the acute (days 1 through 10 post-challenge) and chronic (days 11 through 35) phases of infection. (C and D) At study end-points, tissues collected from survivors were homogenized, diluted, and spread on agar plates to determine bacterial loads. The symbols show data for individual animals; horizontal lines represent the mean total CFU for each group. The survival curves were compared using the Log-rank Mantel-Cox and Gehan-Breslow-Wilcoxon tests and found to be significantly different with *P* values < 0.0001 in both tests. The curves for the PIV5-BatA and PIV5-Tb groups were found to be significantly different with *P* values of 0.0128 (Log-rank Mantel-Cox) and 0.0041 (Gehan-Breslow-Wilcoxon). The curves for the PIV5-Tb and *batA* KO LAS groups were found to be significantly different with *P* values < 0.0001 in both tests. The curves for the *batA* KO LAS and PIV5-BatA groups were significantly different with *P* values of 0.0438 (Log-rank Mantel-Cox) and 0.0368 (Gehan-Breslow-Wilcoxon). Using the Chi-square (and Fisher exact) test, the number of survivors with no detectable bacteria in the lungs was found to be significantly different between the PIV5-BatA and PIV5-Tb groups (*P* = 0.0455), and between the *batA* KO LAS and PIV5-Tb groups (*P* = 0.0022).

To evaluate the breadth of protection elicited by the platform, mice vaccinated with PIV5-BatA were challenged with the *B. pseudomallei* wild-type strain K96243. The data in Fig. 5A and 5B demonstrate that the vaccine provided 80% and 60% protection against death during acute and chronic infection, respectively. Vaccination with the PIV5-Tb control virus resulted in 33% and 20% survival during the acute and chronic stages of disease, respectively. Consistent with our previously published findings [19], immunization with the *batA* KO LAS afforded excellent levels of protection against mortality throughout the duration of the experiment.

Analysis of the bacterial burden in the lungs and spleen of mice that survived *B*. *pseudomallei* challenge shows that no bacteria could be detected in the lungs of *n* = 7 out of 9 mice immunized with PIV5-BatA (Fig. 5C). We also found that *n* = 4 survivors from the PIV5-BatA vaccine group had completely cleared the organism from the spleen (Fig. 5D, *n* = 2 mice given the vaccine had no detectable bacteria in the lungs or spleen). All surviving animals immunized with control PIV5-Tb virus were colonized with *B. pseudomallei* in the lungs and spleen (Fig. 5C and 5D, respectively). All *n* = 12 survivors vaccinated with LAS had cleared bacteria from the lungs (Fig. 5C) and *n* = 7 of these protection benchmark animals had no detectable organism in the spleen (Fig. 5D). Taken together, the results demonstrate that a single dose of PIV5 expressing BatA provides excellent survival against *B. pseudomallei* lethal aerosol challenge. Vaccination with PIV5-BatA also resulted in complete bacterial clearance from lungs and spleen of 78% and 44% of survivors, respectively.

**Fig. 6:**
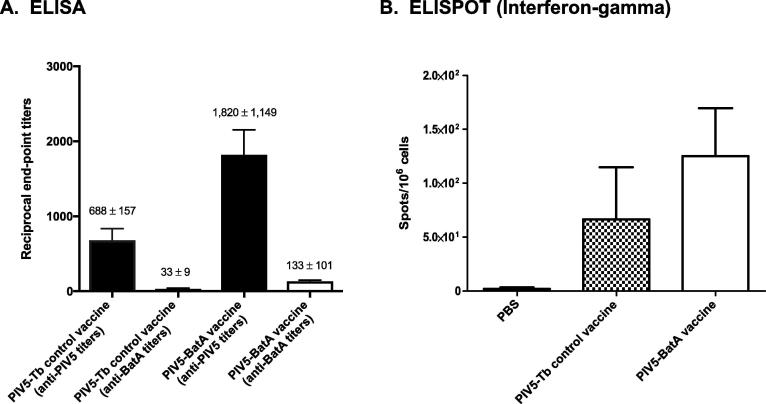
*Humoral and cellular immune responses in mice vaccinated with PIV5-BatA*. (A) Individual serum samples from mice immunized with 10^7^ PFU of PIV5-Tb control vaccine and PIV5-BatA were serially diluted, placed in duplicate wells of plates coated with 10^6^ PFU of PIV5 virions (anti-PIV5 titers) and His-tag BatA protein, and tested by standard ELISA. Alkaline-phosphatase-conjugated goat anti-mouse antibodies specific for the heavy and light chains of IgG were used for detection. The data are expressed as the mean (±standard error) end point titer of samples from *n* = 45 animals. Individual titers were determined using pre-immune samples as background, and correspond to the highest immune serum dilutions giving ELISA values greater than the mean value of pre-immune serum plus 3 standard deviations. (B) ELISPOT analysis of splenocytes from immunized mice. Spleens from *n* = 5 mice were collected were processed and stimulated with purified BatA protein.

To gain information regarding antibody responses elicited by the PIV5-BatA vaccine, serum samples were tested by ELISA and showed antibody titers against PIV5 antigens but little to no reactivity with the BatA protein (Fig. 6A). Cellular immune responses were also examined using an IFN-g ELISPOT assay and the results indicate that splenocytes stimulated with the BatA protein produce higher levels of the cytokine (Fig. 6B).

## 4 Discussion

There has been significant effort in the past decade to devise countermeasures for glanders and melioidosis. However, many challenges remain including considerable gaps understanding immune mechanisms and correlates of protection, the identification, characterization and validation of protective antigens, and the availability of efficient vaccine delivery platforms [24], [25], [26]. *Burkholderia* LAS have thus far provided the most robust protection, but safety concerns hinder their eventual use in humans. *Burkholderia mallei* and *B. pseudomallei* cause life-threatening diseases that are difficult to treat and one would need to ascertain that reversion to virulence is not possible. The organisms can also persist *in vivo* for extended periods, the longest reported prior to appearance of symptoms being 62 years [47]. Hence, there is concern that *Burkholderia* LAS might establish chronic infection, especially in immunocompromised individuals (a growing population in today’s society and risk group for glanders and melioidosis). Capsule [48], [49], [50], LPS [20], [21], [51], and outer membrane vesicles [22], [23], [52], [53] have been investigated as subunit vaccines and showed excellent protection against acute lethal challenge. However, production of these vaccines requires large-scale culture of pathogenic organisms under BSL3 containment and conjugation of capsule and LPS to a carrier to optimize immunogenicity. These processes are costly and hazardous. The use of recombinant proteins in subunit vaccines has shown some promise, but also presents challenges including protein purification and folding, restricted immunogenicity, formulation of multivalent candidates, and the need for adjuvants and multiple boosts to achieve protection [24], [25], [26]. A consistent finding with best-in-class subunit vaccines and *Burkholderia* LAS tested in mice is the ability to induce protection against acute lethal challenge, as evidenced by increased time-to-death, but the inability to achieve complete protection and sterile immunity. A significant number of vaccinated animals develop granulomas and lesions in target tissues with high bacterial burden and eventually die of chronic disease. The failure of current experimental vaccines to eliminate infection clearly represents a major obstacle.

In an effort to advance glanders-melioidosis vaccine development, we introduced both a new antigen and vaccination technology to the field. Our results demonstrate that the autotransporter BatA is a valuable target for developing countermeasures against both *B. mallei* and *B. pseudomallei*. We also establish PIV5 as a novel vaccine delivery platform for the organisms that evokes cross-species protection against lethal aerosol exposure. PIV5 is a non-segmented negative strand RNA virus of the family *Paramyxoviridae* and is thought to be a contributing factor of kennel cough [37], [54]. Several characteristics make it an excellent vector for developing countermeasures. Kennel cough vaccines containing live PIV5 have been used for more than 30 years with an excellent record of safety and efficacy. Humans are exposed to the virus due to close contact with vaccinated dogs. Approximately 30% of the population has neutralizing antibody titers against PIV5, yet no recorded illnesses have been attributed to the virus [55]. Thus, PIV5 is safe and elicits immune responses in humans. PIV5 is safer than other viral vaccine vectors such as adenovirus and poxvirus because it does not have a DNA phase in its replication cycle. Hence, the possible unintended consequences of genetically modifying host DNA through virus recombination and/or insertion are avoided. The genomic structure of PIV5 is stable, as demonstrated by recombinant expression of GFP throughout 10 consecutive passages (10^100^-fold expansion) of the virus [56]. Therefore, PIV5 is better suited than positive strand RNA viruses (*e.g.* alphavirus) as a vaccine delivery system because the genomes of the latter can recombine and rapidly delete inserted genes. Unlike icosahedral viruses, PIV5 virions possess many shapes and forms [37], [54]. This pleomorphic structure provides flexibility to accommodate changes in the size of the genome incurred by the insertion of genes encoding vaccine targets; inserts up to 5-kb have been successfully cloned by our group without affecting virus growth or the integrity of virions (data not shown). PIV5 infects a wide range of cell types, including human primary cells, and induces negligible cytopathic effects [37], [54], [57]. It also infects most mammals and elicits immune responses without causing disease [37], [54], [58], [59], [60]. Thus, PIV5 can be safely used as a vaccine platform in a wide range of hosts and is well-suited for the One Health approach to develop countermeasures against diseases transmitted from animals to humans such as glanders and melioidosis. PIV5 can be grown for several weeks in common cell lines including Vero cells, which are approved for vaccine production, while virions are continuously released into the culture medium at high titers [37], [54], [61]. This provides a scalable, cost-effective, FDA-approved, clear path to vaccine production. Another attribute of the PIV5 vaccination system is its flexibility. The platform is amenable to development of multivalent vaccines and allows for rapid response to new emerging pathogen strains expressing antigenically variable (and/or new) targets, which can be readily introduced in the recombinant PIV5 virus vaccine production pipeline. Importantly, previous work by our group demonstrate that single dose immunization is sufficient to provide excellent protection against many infectious agents, and that pre-existing immunity against PIV5 does not block the vector’s ability to boost immune responses against recombinantly-expressed vaccine targets [55], [62], [63], [64].

The exact mechanism of protection afforded by the PIV5-BatA vaccine is not clear at this time. The analysis of serum samples from vaccinated mice shows little to no antibody titers against BatA and IFN-g ELISPOT data indicate that spleen cells stimulated with the protein produce higher levels of the cytokine. These findings suggest that antibodies do not play a predominant role in protection and that T cell responses are primarily responsible for clearance of *B. mallei* and *B. pseudomallei* from target tissues, which is consistent with the ability of the organisms to thrive intracellularly. It is also possible that intranasal vaccination with PIV5-BatA elicits robust mucosal antibody responses against the autotransporter, which in turn interfere with the ability of *B. mallei* and *B. pseudomallei* to establish themselves in the respiratory tract upon aerosol challenge and persist in the host.

In conclusion, our study demonstrates that vaccination with recombinant PIV5 virus expressing the highly-conserved *Burkholderia* antigen BatA elicits excellent protection against death upon aerosol exposure to *B. mallei* and *B. pseudomallei* and results in a high proportion of survivors with no detectable *B. pseudomallei* bacteria in the lungs. To our knowledge, this level of protection against aerosol infection by both *B. mallei* and *B. pseudomallei* using only one dose of a single-antigen vaccine has not been reported in the field. Future work comparing and contrasting the kinetics, quality, levels and functionality of immune responses evoked by vaccination with PIV5-BatA prior to and during infection with wild-type *Burkholderia* strains will identify key components of the immune system associated with protection and guide efforts for the rationale design of PIV5-based countermeasures targeting BatA and/or other established immunoprotective antigens, with the goal of achieving sterilizing immunity against glanders and melioidosis.
